# The Influence of Orthography on Phonemic Knowledge: An Experimental Investigation on German and Persian

**DOI:** 10.1007/s10936-019-09664-9

**Published:** 2019-08-19

**Authors:** Leila Nayernia, Ruben van de Vijver, Peter Indefrey

**Affiliations:** 1 Abteilung für Allgemeine Sprachwissenschaft, Institut für Sprache und Information, Heinrich Heine University Düsseldorf, Universitätsstr. 1, 40225 Düsseldorf, Germany; 2 Centre for Cognitive Neuroimaging, Donders Institute for Brain, Cognition, and Behaviour, Radboud University Nijmegen, Nijmegen, The Netherlands; 3 Max Planck Institute for Psycholinguistics, Nijmegen, The Netherlands

**Keywords:** Phonological processing, Phonetic representations, Phonological awareness, Orthography, Grapheme, Orthographical influence, Auditory processing of phonemes, Persian, German

## Abstract

This study investigated whether the phonological representation of a word is modulated by its orthographic representation in case of a mismatch between the two representations. Such a mismatch is found in Persian, where short vowels are represented phonemically but not orthographically. Persian adult literates, Persian adult illiterates, and German adult literates were presented with two auditory tasks, an AX-discrimination task and a reversal task. We assumed that if orthographic representations influence phonological representations, Persian literates should perform worse than Persian illiterates or German literates on items with short vowels in these tasks. The results of the discrimination tasks showed that Persian literates and illiterates as well as German literates were approximately equally competent in discriminating short vowels in Persian words and pseudowords. Persian literates did not well discriminate German words containing phonemes that differed only in vowel length. German literates performed relatively poorly in discriminating German homographic words that differed only in vowel length. Persian illiterates were unable to perform the reversal task in Persian. The results of the other two participant groups in the reversal task showed the predicted poorer performance of Persian literates on Persian items containing short vowels compared to items containing long vowels only. German literates did not show this effect in German. Our results suggest two distinct effects of orthography on phonemic representations: whereas the lack of orthographic representations seems to affect phonemic awareness, homography seems to affect the discriminability of phonemic representations.

## Introduction

By learning to read and write an alphabetic writing system, a connection is made between constituents of written words (letters/graphemes) and the constituents of the spoken forms of words (sounds/phonemes) in memory. A number of researchers have claimed that written constituents can affect the way the spoken constituents are perceived and processed (see e.g. Ehri 2014; Kolinsky 2015). Such effects might not be prominent in transparent orthographies where the grapheme-phoneme mapping across words is consistent. However, opaque orthographies, in which the correspondence between graphemes and phonemes is incomplete or inconsistent, provide a good opportunity to find how a mismatch or inconsistency between written and spoken constituents might influence the perception/production and processing of spoken items.

For example, English speakers hear one more sound in the orally presented words “catch” and “badge” compared to “much” and “page”, respectively (Ehri and Wilce 1980); and children in fourth grade have problems identifying the phoneme /f/ in ‘laughter’ but not in ‘rafter’ (Castles et al. 2003). In rhyme judgment, words such as “broom-room” were judged faster than those spelled differently (such as “tomb-room”) (Seidenberg and Tanenhaus 1979). Orthography can even change the number of perceived syllables of a word. For example, speakers who knew the correct spelling of the word “interesting” perceived one more syllable in it than those who spelled it as “intresting*” (Ehri 1987). Further effects of orthography on explicit phonemic processing have been shown in tasks such as phoneme addition and deletion (Mann and Wimmer 2002; Caravolas and Bruck 1993), phonological length judgment (Cassar and Treiman 1997), and rhyme detection (Prakash et al. 1993).

Other studies have found that inconsistency of spelling has an effect on speech perception in an auditory lexical decision task (Pattamadilok et al. 2010) and in auditory word recognition (Ziegler et al. 2008; Pattamadilok et al. 2007; Ziegler et al. 2004). For example, spoken words could be recognized faster when they were primed by words that had the same initial phonological and orthographic form: message-mess. By contrast, words that shared the same initial phonological form, but have different spelling compared to the prime (definite-deaf) were recognized more slowly (Chereau et al. 2007; Jakimik et al. 1985).

Orthography can affect word production as well (Saletta et al. 2016; Rastle et al. 2011). Saletta et al. (2016), for example, found that orthographic transparency had an effect on auditory pseudoword repetition in adults and children. Non-transparent pseudowords, i.e. pseudowords that could have multiple spellings (e.g. /fispet/ which could be spelled <feespait> or <feespaight>) were produced less accurately, as compared to transparent pseudowords. The repetition ability was measured after a reading task. The accuracy of pseudoword repetitions correlated with the reading level of the participants. The lower the reading proficiency, the less accurate was the repetition of nontransparent pseudowords. The authors concluded that reading and speaking strategies were not the same for proficient and less proficient readers.

Orthography even has an effect on the memory performance for words. In a word form preparation paradigm, Damian and Bowers (2003) found that words from a ‘homogeneous phonology-orthography’ block could be remembered better than words from a ‘homogeneous phonology-heterogeneous orthography’ block, when cued after a memorization phase. As such ‘camel’ was recalled better in a ‘camel-coffee’ block than in a ‘camel-kidney’ block, when cued by the second words. Similarly, Baluch and Danaye-Tousie (2006) found that opaque (non-transparent) Persian words (e.g. <kmk> /komӕk/, ’help’) were recalled less accurately as compared to transparent words (e.g. <satur> /sɑtur/, ‘hatchet’).

In contrast to these studies supporting an effect of orthography on speech processing, Ventura and colleagues (2004, 2007) found no effect of orthography in a shadowing task. These authors, therefore, suggested that there is no effect of orthography on pre-lexical processes and that such effects are limited to lexical processes. There are also authors who claim that the effect of orthography on speech processing may be limited to decision processes or the strategic deployment of orthography (see also Cutler and Davis 2012; Cutler et al. 2010).

In most studies on effects of orthography on speech processing, polygraphic (a phoneme is represented by more than one grapheme, such as /s/ represented by <c> and <s>), polyphonemic (one grapheme represents more than one phoneme, such as <c> which represents both /s/ and /k/), or silent graphemes (a grapheme has no phonemic correspondence such as <t> in “catch”) have been investigated. In the present study, we targeted the effect of orthographically unrepresented phonemes on speech processing. Orthographically unrepresented phonemes are found in the conventional writing system of Persian. Persian is an Indo-European language with a borrowed Semitic script which replaced Pahlavi script in the year 651 after the islamization of Persia (Hayden 2018). The conventional written form does not cover short vowel phonemes that are present in the spoken language; hence, the mapping between phonology (phoneme) and orthography (grapheme) can be considered as incomplete in this respect. An unconventional transparent form of the Persian script in which all sounds are orthographically represented is only used in the early months of learning to read and write (6–8 months) after which it is replaced by the conventional form. Persian, therefore, provides a good opportunity to investigate how speech might be processed differently due to long exposure to orthographically unrepresented phonemes in the conventional writing system of Persian.

In the Persian conventional writing system, short vowel phonemes /ӕ/, /e/, and /o/ are not represented; whereas, the long vowels^1^ /ɑ/, /u/, and /i/ have orthographic representations in both the conventional and the unconventional form. For example,  <par> /pӕr/ (feather) in unconventional writing becomes  <pr> /pӕr/ (feather) and is homographic with  <pr> /por/ (full) in conventional writing. By contrast,  <pir> /pir/ (old) contains one long vowel that is represented in both the conventional and the unconventional forms. Considering the effect of orthography on speech processing, it can, therefore, be hypothesized that items with short vowel phonemes should be processed poorly by monolingual, literate Persian speakers after a long exposure to the conventional writing form.

We investigated this effect in adult Persian speakers by means of two tasks, an implicit AX discrimination task and an explicit phoneme reversal task. In addition to Persian literate participants, we included two other participant groups for comparison: Persian illiterate adults as a group of speakers who possess phonological but no orthographic representations, and German literate participants (adults) who have been exposed to a script that represents both short and long vowels in its phonology and orthography. Insofar as speech processing is modulated by orthographic features after long exposure to a writing system, we expected that Persian literates (but not Persian illiterates and German literates) to perform significantly more poorly where the processing of items with short vowels is required.

By using two tasks that differed with respect to the levels of processing (see Morais 2003 for more information), we aimed to distinguish between possible effects of orthography on the phonemic representations for short vowels at different levels of processing (unconscious and conscious processing). An orthographic influence on the phonemic representations as such would predict a poorer performance of Persian literates in both tasks. By contrast, if orthography is ineffective in prelexical perceptual processing of spoken form (Ventura et al. 2004, 2007), we should find a dissociation of performance in the two tasks, i.e. Persian literates should show unreduced performance in AX-discrimination task and reduced performance in the reversal task compared to the control groups.

## Experiment 1: AX Phoneme Discrimination Task

### Participants

Three groups with 20 participants each took part in the experiment: Persian literates (10 female, mean age 31, age range 20–47), Persian illiterates (14 female, mean age 49, age range 38–60), and German literates (8 female, mean age 30, age range 20–58). For literate participants inclusion criteria were a minimum of 14 years of exposure to written texts and daily reading in their native language. For illiterate participants inclusion criteria were that they had never attended a literacy course and were unable to read simple texts (children’s books) or name the sounds corresponding to Persian letters presented to them in an informal pre-test. All participants were required to have unimpaired hearing/motor response/speech production.

Persian participants were all from the same region in Iran (Fars province): literates from two major cities, illiterates from a smaller town nearby. German participants were all from the Rhine area. All participants signed an informed consent form and were paid a small sum (8 €) for their participation.

### Stimuli

We prepared two lists: one contained pairs of German pseudowords or words; the other contained pairs of Persian pseudowords or words. For both languages, pseudowords were created by changing at least one phoneme of a real word and assessed by three native speakers as being possible but non-existing words. Each list was spoken by a female native speaker clearly and fluently in the standard language. The (pseudo)words of a pair were either identical, such as ‘/korb–korb/’ (<Korb>, ‘basket’) in German, and ‘/mɑst-mɑst/’ (<mast>, ‘Yoghurt’) in Persian; or non-identical, differing in one vowel phoneme such as ‘/gold-geld/’(<Gold> ‘gold’, <Geld> ‘money’) in German and ‘/mehr-mohr/’ (<mhr>, ‘affection’, ‘stamp’) in Persian. Ten warm-ups were included at the beginning of each list.

In eight different conditions (see Table 1 for an overview), non-identical (pseudo)words of a pair differed in one short vowel (S), long vowel (L), or in vowel phonemes with different length (mixed) _ (pseudo) minimal pairs. In the ‘mixed’ conditions, the (pseudo)words were either homographic (e.g. /xod-xud/ < xud>, ‘self/own’- ‘helmet’ in Persian and /su:xt-sƱxt/ <Sucht-sucht> ‘addiction’-(he) ‘searches’ in German) or heterographic (e.g. as ‘/bæxt - bɑxt/ <bxt-baxt> ‘fate’-‘loss’ in Persian and /wo:nən-wɔnən/ <wohnen-Wonnen> ‘to live’-‘pleasures’ in German). Although pseudowords, by definition, do not have any lexicalized orthographic representations, we assumed that literate participants might visualize the written forms of pseudowords while discriminating non-identical pseudo minimal pairs. The pseudo minimal pairs that could have identical orthographic representations (e.g. /ʃantən-ʃa:ntən/ written as <schanten> in German) according to the grapheme-to-phoneme correspondence rules of the respective language were considered as (potentially) ‘homographic’. The homographic pseudo minimal pairs contrast the heterographic pseudo minimal pairs (e.g. /ʃpal-ʃpa:l/ <spall - spal> in German) in the sense that the pairs could have different orthographic forms.

**Table 1:** Levels of condition and examples in discrimination task

Condition	Abb.	German				Persian			
		Example	Written	No. of stimuli	Mean no. of phonemes	Example	Written	No. of stimuli	Mean no. of phonemes
Long vowel pseudo word	L-P	/ʃta:n-ʃtu:n/	Stahn-Stuhn	13	4.46	/lik-luk/		12	3.17
Short vowel pseudo word	S-P	/broŋ-brɛŋ/	Brong-Breng	13	4.85	/pӕŋ-pɛŋ/		13	3.69
Long vowel real word	L-R	/ʃtu:l-ʃti:l/	Stuhl-Stil chair-style	13	3.77	/fɑl-fil/	fortune telling - elephant	13	3
Short vowel real word	S-R	/hɛft-haft/	Heft-Haft booklet-arrest	13	4.23	/ʃӕk-ʃok/	doubt - shock	13	3.61
Mixed vowel homograph pseudo word	Mixed-H-P	/ʃantən-ʃa:ntən/	Schanten-schanten	13	5.92	/douf-duf/		13	2.92
Mixed vowel pseudo word	Mixed-P	/ʃpa:l-ʃpal/	Spahl-Spall	13	4.46	/pӕrm-purm/		13	3.61
Mixed vowel homograph real word	Mixed-H-R	/ra:stə-rastə/	raste–raste (I)rest-(he)raced	13	4.46	/xod-xud/	self/own - helmet	13	2.77
Mixed vowel real word	Mixed-R	/ʃlaf-ʃla:f/	schlaff-Schlaf flaccid-sleep	13	4.38	/bӕxt-bɑxt/	fate - loss	13	3.15

The lists contained 13 non-identical (pseudo)word pairs per condition (in total 104 non-identical pairs) as well as 52 pairs of identical (pseudo)words with long vowels and 52 pairs of identical (pseudo)words with short vowels. The order of (pseudo)words in a pair was counterbalanced across participants. The order of the (pseudo)word pairs in the list was pseudo-randomized across participants with the constraints that (a) no more than two consecutive trials should have the same expected response (‘identical’ or ‘different’) and (b) conditions should not be repeated in consecutive trials.

### Procedure

The participants were asked to decide by a button press whether two spoken (pseudo)words were identical or different. Each participant was tested alone in a quiet room. A short beep sound signaled a new trial. One hundred milliseconds after the beep sound, a trial consisting of 2 successive spoken (pseudo)words was played through a headset (Sennheiser SC165). The inter-stimulus-interval between the (pseudo)words within a trial was 200 ms. After the onset of the second (pseudo)word the participants had 2000 ms to press the dedicated buttons for ‘identical’ or ‘different’ responses.

For German and Persian literate participants, the AX phoneme discrimination task session had two parts of 20 min with a short break in between. In the first part, half of the participants performed the discrimination task in their native language; the other half performed the discrimination task in the unknown language. In the second part the assignment of languages was reversed. Illiterate participants only performed the discrimination task in Persian.^2^

The AX phoneme discrimination experiment was programmed in Psychopy V1.82.01 (Peirce et al. 2019) and run on a Sony laptop. The stimuli were played via a headset and participants pressed the left or right arrow keys (buttons) on the keyboard of the laptop for ‘identical’ and ‘different’ responses. The button assignments were reversed for half of the participants.

### Results

**Fig. 1:**
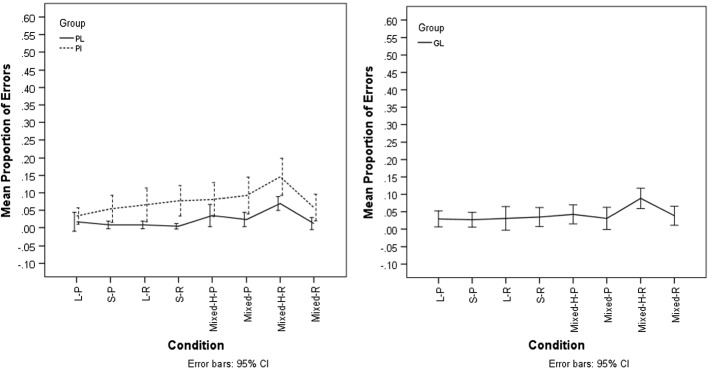
Discrimination in Persian. Left panel: mean proportion of errors for Persian literate (PL) and illiterate (PI) participants. Right panel: mean proportion of errors for German literate participants (GL). (*L* long vowels;* S* short vowels, * H* homographic,* P* pseudoword,* R* real word)

**Fig. 2:**
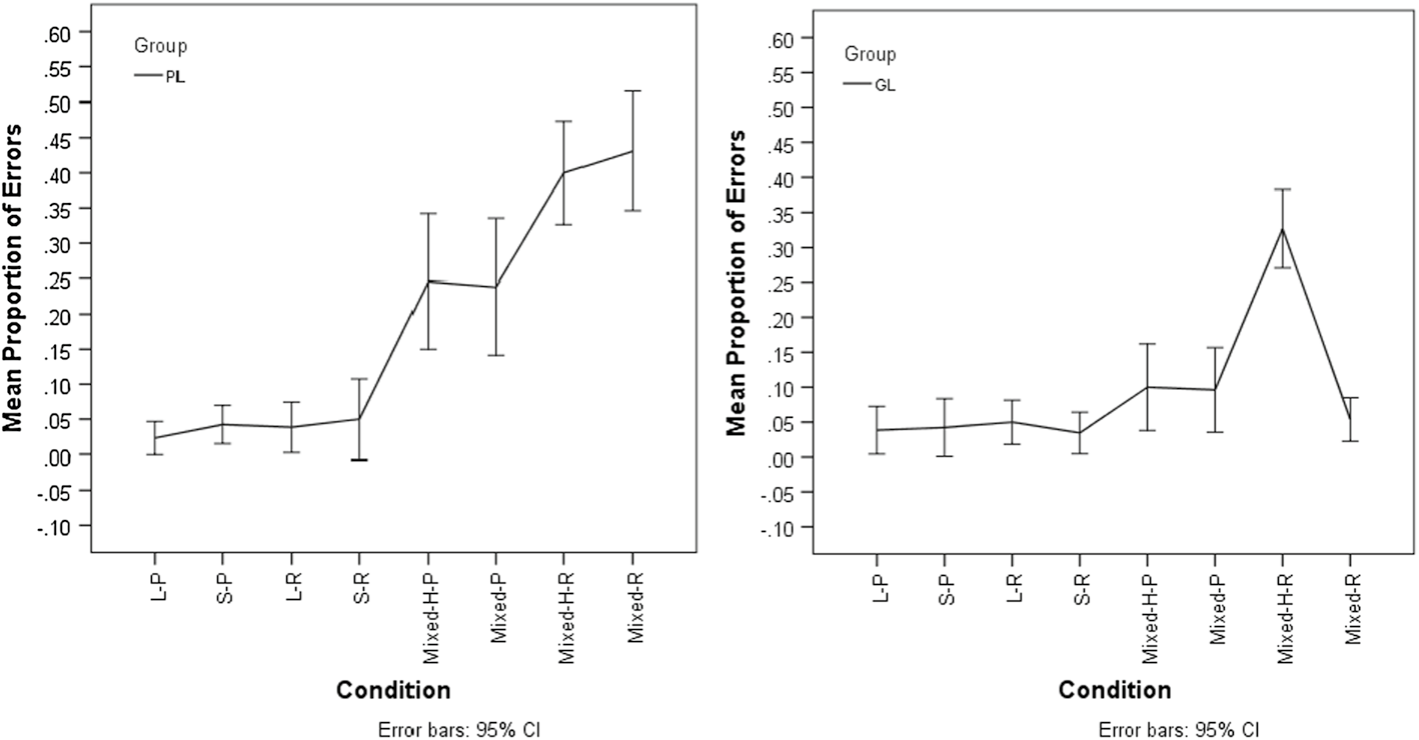
Discrimination in German. Left panel: mean proportion of errors for Persian literate participants (PL). Right panel: mean proportion of errors for German literate participants (GL). (Abbreviations: L = long vowels; S = short vowels; H = homographic: P = pseudoword; R = real word)

The proportions of errors (incorrect responses and no responses) per condition are presented in Figs. 1 and 2 (graphs were generated using SPSS 21, IBM Corp 2012). We used the lme4 package of R (Bates et al. 2015; R Core Team 2014) to perform general linear mixed effects regression analyses with the dependent variable ‘accuracy’ (correct, incorrect) and the predictor variables ‘Literacy’ (literate, illiterate) and ‘Condition’.^3^ As performance on Persian and German tasks could not be meaningfully compared for Persian and German participants, we conducted separate analyses for the two languages. Both predictor variables were entered into the model as fixed effect(s) for Persian participants discriminating Persian minimal pairs. In all other models ‘Condition’ was the only fixed factor. As random effects, the intercepts for ‘participant’ and ‘word’ as well as by-participant random slopes for the effect of Condition were entered into the model. The “multcomp” package (Tukey test) (Hothorn et al. 2008) was used to run post hoc pair-wise comparisons between different levels of the fixed factor(s). *p* values were obtained by likelihood ratio tests of the full model with the effect in question against the model without the effect in question.

#### Discrimination in Persian

##### Persian Participants

As shown in Fig. 1, Persian illiterates made about twice as many errors (error proportion range 0.03–0.15) as Persian literates (error proportion range 0.0–0.07) across all levels of Condition when discriminating Persian (pseudo)minimal pairs. For both groups, the proportion of errors was the highest when the two stimuli were homographic real words containing vowels of different lengths.

Results of a general linear mixed effects analysis with the dependent variable ‘Accuracy’ and the predictor variables ‘Condition’ and ‘Literacy’ showed that both predictors had significant effects [Condition: *X*^2^(7) = 18.6, *p *= 0.009; Literacy: *X*^2^(1) = 15.85, *p *= 0.000]. The interaction between the two predictors was not significant [*X*^2^(7) = 5.54, *p* = 0.59]. Tukey-corrected pair-wise comparisons between the levels of the predictor Condition showed no significant differences (all *p*s > 0.11).

##### German Participants

For German literates discriminating Persian word and pseudoword pairs, the proportions of errors ranged from 0.03 to 0.09. Like for the Persian participants, the proportion of errors was highest when the two stimuli were homographic real words containing vowels of different lengths.

Results of a general linear mixed effects analysis with the dependent variable ‘Accuracy’ and the predictor variable ‘Condition’ showed a marginally significant effect of the predictor Condition [*X*^2^(7) = 14.022, *p *= 0.051]. Tukey-corrected pair-wise comparisons between the levels of the predictor Condition showed no significant differences (all *p*s > 0.36).

#### Discrimination in German

##### Persian Participants

As shown in Fig. 2, Persian literates had no problems discriminating German words or pseudowords containing different long vowels or different short vowels (error proportion range 0.2–0.5). By contrast, Persian literates performed much poorer in the ‘mixed’ conditions, i.e. on German words or pseudowords that differed only in vowel length (error proportion range 0.24–0.43).

Results of a general linear mixed effects analysis with the dependent variable ‘Accuracy’ and the predictor variable ‘Condition’ showed that the predictor Condition had a significant effect on the accuracy of Persian literate participants discriminating German minimal pairs [*X*^2^(7) = 52.59, *p* = 0.000]. Tukey-corrected pair-wise comparisons between the levels of the predictor Condition showed that the performance of Persian literates on homographic and heterographic real words of mixed vowel lengths was significantly lower than their performance on words and pseudowords with homogeneous vowel length (all *p*s ≤ 0.05). No other pair-wise comparison reached significance (all *p*s > 0.07).

##### German Participants

German literates performed as well as Persian literates discriminating German words or pseudowords containing different long vowels or different short vowels (error proportion range 0.3–0.5). The proportion of errors in the mixed conditions was higher (error proportion range 0.05–0.33) with a particularly high error rate for homographic real words that differed only in vowel length (0.33). By contrast, the error rate for non-homographic real words that differed only in vowel length was comparatively low (0.05).

Results of a general linear mixed effects analysis with the dependent variable ‘Accuracy’ and the predictor variable ‘Condition’ showed that the predictor Condition had a significant effect on the accuracy of German literate participants discriminating German minimal pairs [*X*^2^(7) = 37.704, *p* = 0.000]. Tukey-corrected pair-wise comparisons between the levels of the predictor Condition confirmed that the performance on homographic real words that differed only in vowel length was significantly different from all other conditions (all *p*s < 0.025). No other pair-wise comparison reached significance (all *p*s > 0.77).

### Discussion

Experiment 1 provided no evidence for an influence of the lack of short vowel graphemes on the processing short vowels. Persian literates as well as the two control groups performed equally well on Persian words/pseudowords containing short vowels as they did on words/pseudowords containing only long vowels. One possibility for the absence of a vowel length effect could be that the task as such did not tap into phonemic representations at all. The participants might have discriminated the input stimuli solely based on their acoustic representations.

A number of findings of Exp. 1 speak against this possibility. Firstly, Persian illiterates performed significantly worse than Persian literates across all stimulus types. Learning to read and write affects phonemic rather than acoustic representations of words and there is evidence that phoneme boundaries may be sharpened by literacy (for a review see Kolinsky 2015). Hence, the observed difference between the two groups suggests that the participants accessed phonemic representations for discrimination and these representations enabled literate participants to perform better.

Secondly, we observed an unpredicted influence of orthography in German participants performing the same task with German materials. These participants performed significantly worse on homographic word pairs compared to all other conditions suggesting that identical orthographic representations interfere with auditory discrimination. We will come back to this finding in more detail in the general discussion; but as far as the kind of representations is concerned that are accessed in order to perform our discrimination task, it seems more likely that orthographic representations interact with phonemic representations rather than acoustic representations.

Finally, Persian literates had great difficulties discriminating German words and pseudowords that differed only in vowel length (as in <Schote-Schotte>, /ʃo:tə-ʃɔtə/). Such a contrast is not phonemic in Persian (Tsukada 2011; Campbell and King 1991) whereas it is phonemic in German (Steinbrink et al. 2012) suggesting that the high error rate of Persian listeners is due to the fact that they did not perceive a phonemic difference between these items.

Based on these considerations, we assume that the participants performed our discrimination task by comparing phonemic representations of the input stimuli at least in addition to acoustic representations. The absence of a vowel length effect in Persian literates could, thus, mean that the lack of short vowel graphemes has no effect on phonemic representations. Alternatively, the lack of short vowel graphemes may not affect perceptual phonemic processing, but does affect processes requiring phonemic awareness. In order to distinguish between these two options, we will now turn to Experiment 2 that used a task that required phonemic awareness.

## Experiment 2: Reversal Task

### Participants

The same participants as in Experiment 1 were asked to participate in the phoneme reversal task in their native language. Despite being trained on the task for at least 30 min, Persian illiterates turned out to be unable to perform the task.

### Stimuli

45 disyllabic Persian and 35 disyllabic German words with cvcvc syllable structure were used as stimuli for the Persian and German reversal tasks (see Table 2). The difference in the number of stimuli was due to the shortage of suitable stimuli in German.

**Table 2:** Stimuli and conditions in phoneme reversal task

Vowel category	Abbreviation	Persian			German		
Levels		No. of stimuli	Example	Written	No. of stimuli	Example	Written
Short vowel–short vowel	S	11	/pɛsӕr/	boy	8	/balɛt/	Ballett ballet
Long vowel–long vowel	L	11	/diruz/	yesterday	6	/t͡suːtaːt/	Zutat ingredient
Short vowel–long vowel	Mixed	23	/muʃӕk/	missile	21	/da.tƱm/	Datum date

The words were selected so that either all sounds in a word were different (c1v1c2v2c3) or the initial and final sounds were identical and the rest were different (c1v1c2v2c1). According to the distribution of the vowel lengths within words, the stimuli fell into different conditions: both vowels of the word (v1 and v2) were short vowels (S), both vowels of the word were long vowels (L), or one of the vowels of the word was short and the other long (mixed). In the ‘mixed’ condition the two vowels differed both in length and vowel quality. In approximately half of the stimuli of this condition a long vowel was followed by a short vowel. In the other half the order was reversed. All of the words were real words of the respective language and their reversal always resulted in a pseudoword.

The order of the stimuli was pseudo-randomized within lists with the constraint that stimuli from the same condition did not immediately follow each other. Each participant received a different list. All words were spoken clearly and with a normal tempo in the standard form of the respective language by the same speakers as in the discrimination task.

### Procedure

The phoneme reversal experiment was programmed in Psychopy (Peirce et al. 2019). Each participant was tested in a separate session following a short break after the discrimination task. The experimental task along with some examples was explained to the participants.

An experimental trial started with a beep sound. 200 ms later, the spoken stimulus was played through a headset (Sennheiser SC165). From the onset of the stimulus, the participants had 5000 ms to reverse the phonemes of the word, and speak the reversed form into the microphone of the headset. In total, the experiment took about 5 min. The participants’ responses were recorded as audio files on a laptop and analyzed with Praat software (Boersma and Weenink 2015). The responses were manually transcribed and coded as ‘correct’ or ‘incorrect’ by the first author. We considered responses as correct when the two vowels were reversed. Incorrect responses included failures to reverse the vowels, replacements of one or both of the vowels by other vowels as well as incomplete responses or failures to respond altogether.

### Results

**Fig. 3:**
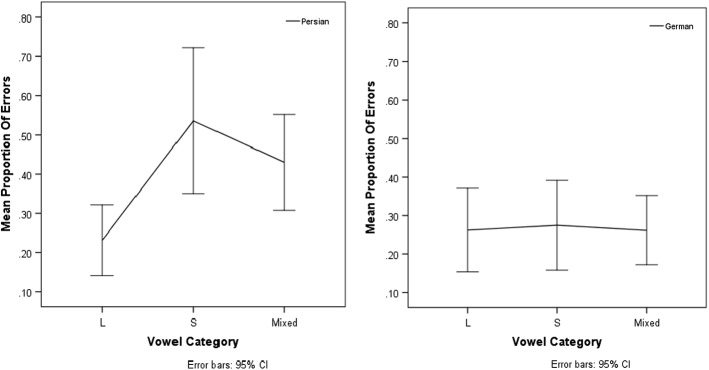
Reversal task. Left panel: mean proportion of errors for Persian literate participants. Right panel: mean proportion of errors for German literate participants. (Abbreviations: L = two long vowels; S = two short vowels; Mixed = one long vowel and one short vowel)

The proportions of errors per condition are presented in Fig. 3. General linear mixed effects regression analyses with the dependent variable ‘accuracy’ (correct, incorrect) and the predictor variable ‘Vowel category’ (two long vowels, two short vowels, mixed) were conducted in the same way as for Experiment 1.

#### Persian Participants

As shown in Fig. 3, Persian literates made more reversal mistakes on Persian words containing at least one short vowel compared to words that contained two long vowels (proportion of errors: two short vowels 0.54; mixed 0.43; two long vowels 0.23). The main error types were vowel changes (46.7% of all errors), failures to reverse (i.e. reproducing the original stimulus, 24.2%), and ‘no reply’ (17.2%).

Results of a general linear mixed effects analysis (Bates et al. 2015; R Core Team 2014) showed that the predictor Vowel Category had a significant effect on the reversal accuracy of Persian literate participants [*X*^2^(2) = 12.9, *p* = 0.002]. Tukey-corrected pair-wise comparisons (Hothorn et al. 2008) between the levels of the predictor Condition confirmed that the performance on words with two long vowels was significantly better than the performance on words containing two short vowels (*Z* = 3.360, *p* = 0.002) and the performance on words containing one long and one short vowel (*Z* = 3.679, *p* = 0.001). The error rates for words that contained two short vowels did not differ significantly from the error rates for words that contained vowels of mixed lengths (*Z* = 1.467, *p* = 0.293).

#### German Participants

For German participants, the mean proportions of reversal errors on German words were similar across the three vowel categories ranging from 0.26 (two long vowels, mixed) to 0.28 (two short vowels). The main error types were ‘no reply’ (45.5% of all errors), incomplete responses (23.3%), and failures to reverse (16.3%). Vowel changes only occurred in 5.4% of all errors. Results of a general linear mixed effects analysis showed that the predictor Vowel Category had no significant effect on the reversal accuracy of German literate participants [*X*^2^(2) = 0.004, *p* = 1].

### Discussion

Experiment 2 showed that the presence of a short vowel in the words had a negative impact on the accuracy of Persian literates in the reversal task. By contrast, for German literates the accuracy of the performance was not affected by the vowel category. In the Persian illiterate group, the ability of breaking the integrity of a word into its phonemes could not be attained by a short training session (Morais et al. 1988). The results thus suggest that the lack of short vowel graphemes affects processes requiring phonemic awareness.

It should be noted that the Persian participants correctly reversed about half of the words that contained one or two short vowels. Furthermore, they never produced phonotactically unacceptable reversed strings (e.g. ccc, ccvc structures). These observations make it unlikely that the Persian participants simply read from the visualized spellings of words containing short vowels.

In vowel change errors, the predominant error type of Persian participants, short vowels were mainly replaced by other short vowels, suggesting that the lack of short vowel graphemes in conventional Persian orthography has a negative effect on Persian literates’ ability to identify, maintain, or manipulate short vowels.

## General Discussion

In two experiments, we investigated the ability of Persian literate participants to discriminate (Exp. 1) and manipulate (Exp. 2) words and pseudowords presented auditorily. We hypothesized that due to their long exposure to a script that does not represent short vowels, these participants might show dissociation between their performance on stimuli containing only long vowels and their performance on stimuli containing short vowels. Depending on whether the lack of short vowel graphemes affects phonemic representations underlying speech perception or phonemic representations that can be consciously accessed and manipulated, we expected a poorer performance on stimuli containing short as compared to long vowels in a perceptual task (AX discrimination, Exp.1), in a task requiring phonemic awareness (reversal tasks, Exp. 2) or in both tasks. For comparison, we also presented two other participants groups with the same tasks: a group of Persian illiterates and a group of German literates. We expected none of these groups to show dissociation between their performance on short and long vowels. Persian illiterates had been exposed to spoken Persian to the same degree as the Persian literates; but, they should not show any influence of orthography. German literates had been exposed to written language to the same degree as Persian literates; but, their script does represent both long and short vowels.

The results of our experiments show a clear pattern. There was no evidence for a detrimental influence of the lack of short vowel graphemes on auditory perception; but, there was dissociation between stimuli with and without short vowels in the phonemic awareness task. In Experiment 1, Persian literates discriminated Persian (and German) stimuli containing short and long vowels equally well, and did not differ in this respect from the two control groups. In Experiment 2, Persian literates performed significantly more poorly on the (phoneme) reversal of words containing short vowels than on the reversal of words containing only long vowels; whereas, German literates showed no such difference. In sum, it seems that when phonemes are orthographically unrepresented, they have deficient consciously accessible and manipulable representations; but, the phonemic representations underlying perception are unaffected. This result is in line with Ventura et al. (2004) who assumed two levels for speech processing: a strictly perceptual level and a post-perceptual level. The perceptual level is influenced by linguistic experience and includes modular operations, such as the perceptual segmentation of acoustic representations. At the post-perceptual level, attention and other sources of knowledge affect speech processing as well.

In the Persian script, words that contain only short vowels are homographic. We, therefore, included homographic words also in the German stimuli used in the auditory discrimination task. Most of these stimulus pairs differed only in vowel length. As an unpredicted finding, German listeners performed significantly worse on these stimuli compared to all other stimulus categories, including non-homographic words differing only in vowel length. Persian listeners performed equally poorly on German homographic and non-homographic words differing only in vowel length (numerically even worse on the latter), excluding the possibilities that (a) homographic words just happen to be acoustically more similar than non-homographic words; or (b) that our German speaker inadvertently pronounced homographic words more similarly. It thus seems that we observed a true orthographic effect on auditory perception in German listeners: homography has a detrimental effect on auditory discrimination.

This observation raises the question why the lack of short vowel graphemes in Persian also resulting in homography did not have the same effect. It might be the case that Persian literates discriminated the stimuli at a strictly perceptual (pre-lexical) level whereas German literates might have performed the discrimination task at a post-perceptual, lexical level. In other words, depending on language, the same task might have been performed in different ways. According to Pattamadilok et al. (2007), the sensitivity to word spelling might be different for literates in different orthographies. They mention two decisive factors in this regard: first, what type of information (identical/redundant or different) is conveyed by written and spoken forms of a language; second, how robust is the connection between spoken and written forms of a language. In Persian, as compared to German, the information that is conveyed by the spoken form is more comprehensive than that presented by the written form (opaque written form with respect to short vowels). This feature might make Persian listeners rely more on the spoken form. Hence, they might go for pre-lexical strictly perceptual processing for which no orthographic effect is expected. German listeners, on the other hand, might rely more strongly on accessing lexical orthographic representations online, because the German script, at least with respect to vowel length is rather transparent; and hence orthographic representations usually help to distinguish between vowels differing in length.

For the relatively few words where in spite of different vowel lengths the orthographic representations are identical, this strategy backfires as now the orthographic representations signal identity where there is in fact a phonemic difference. From a computational perspective, for example in the ‘Bimodal interactive activation model’ of Grainger and Ferrand (1996), this situation would correspond to two distinct phonemic representations that both receive feedback from a shared orthographic representation. In consequence, their activation levels become more similar, resulting in poorer discrimination.

In sum, the results of the present study suggest two distinct effects of orthography on phonemic representations: whereas the lack of orthographic representations seems to affect phonemic awareness, homography may affect the discriminability of phonemic representations.

## Footnotes

1 Letters (among them long vowels) are represented orthographically in different shapes based on their positions in words: in middle and final positions, /i/ is represented as  and  respectively.

2 Performing all three tasks (Persian and German AX discrimination, Persian reversal task) turned out to be very demanding for illiterate participants, taking in total 2 h including the required training on how to perform the tasks. As most of them became bored and asked to quit the session, we decided to only conduct Persian AX discrimination with these participants.

3 Levels of Condition: Long vowels-pseudowords, short vowels-pseudowords, long vowels-words, short vowels-words, mixed vowels-homographic pseudowords, mixed vowels-pseudowords, mixed vowels-homographic words, mixed vowels-words.
